# Glial Cell Line-Derived Neurotrophic Factor Gene Delivery in Parkinson's Disease: A Delicate Balance between Neuroprotection, Trophic Effects, and Unwanted Compensatory Mechanisms

**DOI:** 10.3389/fnana.2017.00029

**Published:** 2017-04-10

**Authors:** Liliane Tenenbaum, Marie Humbert-Claude

**Affiliations:** Laboratory of Cellular and Molecular Neurotherapies, Clinical Neuroscience Department, Center for Neuroscience Research, Lausanne University HospitalLausanne, Switzerland

**Keywords:** GDNF, RET, tyrosine hydroxylase, dopamine transporter, retrograde signaling, neurturin, AAV

## Abstract

Glial cell line-derived neurotrophic factor (GDNF) and Neurturin (NRTN) bind to a receptor complex consisting of a member of the GDNF family receptor (GFR)-α and the Ret tyrosine kinase. Both factors were shown to protect nigro-striatal dopaminergic neurons and reduce motor symptoms when applied terminally in toxin-induced Parkinson's disease (PD) models. However, clinical trials based on intraputaminal GDNF protein administration or recombinant adeno-associated virus (rAAV)-mediated NRTN gene delivery have been disappointing. In this review, several factors that could have limited the clinical benefits are discussed. Retrograde transport of GDNF/NRTN to the dopaminergic neurons soma is thought to be necessary for NRTN/GFR-α/Ret signaling mediating the pro-survival effect. Therefore, the feasibility of treating advanced patients with neurotrophic factors is questioned by recent data showing that: (i) tyrosine hydroxylase-positive putaminal innervation has almost completely disappeared at 5 years post-diagnosis and (ii) in patients enrolled in the rAAV-NRTN trial more than 5 years post-diagnosis, NRTN was almost not transported to the substantia nigra pars compacta. In addition to its anti-apoptotic and neurotrophic properties, GDNF also interferes with dopamine homeostasis via time and dose-dependent effects such as: stimulation of dopamine neuron excitability, inhibition of dopamine transporter activity, tyrosine hydroxylase phosphorylation, and inhibition of tyrosine hydroxylase transcription. Depending on the delivery parameters, the net result of this intricate network of regulations could be either beneficial or deleterious. In conclusion, further unraveling of the mechanism of action of GDNF gene delivery in relevant animal models is still needed to optimize the clinical benefits of this new therapeutic approach. Recent developments in the design of regulated viral vectors will allow to finely adjust the GDNF dose and period of administration. Finally, new clinical studies in less advanced patients are warranted to evaluate the potential of AAV-mediated neurotrophic factors gene delivery in PD. These will be facilitated by the demonstration of the safety of rAAV administration into the human brain.

## Glial cell line-derived neurotrophic factor gene delivery: preclinical findings in animal models

Glial cell line-derived neurotrophic factor (GDNF) has first been shown to protect embryonic dopaminergic neurons *in vitro* (Lin et al., 1993). GDNF and related factors, such as neurturin (NRTN), signal through a multicomponent receptor system consisting of a glycosyl-phosphatidylinositol-anchored receptor, the GDNF family receptor (GFR)-α and Ret tyrosine kinase (Paratcha and Ledda, 2008). GDNF and NRTN preferentially bind to GFR-α1 and GFR–α2, respectively (Sariola and Saarma, 2003). In the absence of Ret, GDNF in complex with GFR-α1 may also interact with heparan sulfate glycosaminoglycans to activate the c-Met receptor tyrosine kinase and to neural cell adhesion molecule (NCAM) which activates the Src-like kinase Fyn and focal adhesion kinase (FAK; Sariola and Saarma, 2003).

Since dopaminergic neurons express both GFRα and Ret in the rodent (Jing et al., 1997; Walker et al., 1998) and human (Walker et al., 1998; Quartu et al., 2007) brain, it was soon hoped that GDNF could have a therapeutic potential for Parkinson's disease (PD) (Tomac et al., 1995). The therapeutic benefit of GDNF and NRTN has been demonstrated in phenotypic, toxin-induced [6-hydroxydopamine (6-OHDA) and 1-methyl-4-phenyl-1,2,3,6-tetrahydropyridine (MPTP)] rodent and non-human primate models of PD (Bilang-Bleuel et al., 1997; Choi-Lundberg et al., 1997; Mandel et al., 1999; Kirik et al., 2000; Kordower et al., 2000; Eslamboli et al., 2003, 2005; Kordower et al., 2006; Gasmi et al., 2007a,b; Ramaswamy et al., 2007; Herzog et al., 2008, 2009; Su et al., 2009). Ret induces the serine/threonine kinase AKT and extracellular signal-regulated kinase (ERK) signaling which mediate pro-survival and neurotrophic activities (Sariola and Saarma, 2003). In favor of this hypothesis, it was shown that the absence of Ret signaling caused progressive degeneration of the nigrostriatal system (Kramer et al., 2007). These data are however, in contradiction with another study suggesting that GDNF is dispensable for dopaminergic neurons survival (Kopra et al., 2015). Since GDNF does not pass the blood-brain barrier, intracerebral gene delivery by stereotaxic injection of viral vectors has been proposed as a method of administration. The therapeutic potential of GDNF gene delivery has been evaluated in different pre-clinical paradigms.

### Neuroprotection vs. neurorestoration

In the neuroprotective paradigm, a viral vector encoding GDNF is administered prior to lesioning the nigro-striatal dopaminergic pathway whereas in the neurorestorative paradigm, the vector is administered after lesioning. While a large number of neuroprotection studies (Bilang-Bleuel et al., 1997; Choi-Lundberg et al., 1997; Mandel et al., 1999; Bensadoun et al., 2000; Kirik et al., 2000; Kordower et al., 2000; Eslamboli et al., 2005; Bartus et al., 2011) have been published, only few works aimed at evaluating a potential neurorestorative effect (Kozlowski et al., 2000; Yang et al., 2009; Tereshchenko et al., 2014). In addition, the latter paradigm can refer to different approaches. Indeed, GDNF administration has been performed either during the progressive phase (Kozlowski et al., 2000) or after stabilization of the lesion (Wang et al., 2002; Zheng et al., 2005; Eberling et al., 2009).

In both paradigms, increased numbers of SNpc dopaminergic neurons stained by antibodies directed against tyrosine hydroxylase (TH), an enzyme of the DA biosynthesis pathway, and attenuation of the motor symptoms have been demonstrated.

### Intranigral vs. intrastriatal delivery

Two delivery sites were used and sometimes combined: the SN (Choi-Lundberg et al., 1997; Mandel et al., 1997; Bensadoun et al., 2000) and the striatum (Bilang-Bleuel et al., 1997; Connor et al., 1999; Kirik et al., 2000) in order to deliver GDNF respectively to the dopaminergic neurons cell soma and at the level of their terminals. In some instances, both approaches were combined (Kirik et al., 2000; Kordower et al., 2000).

Most of the studies performed in the neuroprotection paradigm indicated that when GDNF was administered at the level of the SN, cell bodies were protected but no benefit on motor symptoms was observed (Bilang-Bleuel et al., 1997; Mandel et al., 1999). In contrast, when GDNF was administered into the striatum, both cell bodies and terminals were preserved and motor symptoms were reduced (Kirik et al., 2000). Accordingly, several recent studies have suggested that axonal dysfunction precedes neuronal cell death and is better correlated with clinical symptoms (see further; Burke and O'Malley, 2013; Kordower et al., 2013; Schulz-Schaeffer, 2015).

In one report, the authors succeeded to obtain a protection after intranigral delivery of an adenoviral vector in the neurorestorative paradigm (Kozlowski et al., 2000). Strikingly, in this study, dopaminergic neurons survival was assessed by injecting a retrograde tracer, Fluorogold, into the striatum and counting the number of Fluorogold-positive neurons in the SNpc. By that mean, all neurons which still have nigro-striatal projections proficient for retrograde transport are taken into account. In contrast, in the same study, the number of cells expressing TH in the SNpc was not increased by the GDNF treatment. Other studies reported a lack of matching between TH staining and retrograde Fluorogold labeling of nigrostriatal neurons (Sauer and Oertel, 1994). The loss of the TH marker could indicate that the neurons were dysfunctional but still present and possibly rescuable. Thus, the method to assess neuronal survival and pathway integrity is crucial. Neuronal survival evaluation solely based on TH immunostainings could provide misleading interpretations.

### Perturbations of dopamine homeostasis by excessive GDNF overexpression

**Figure 1:**
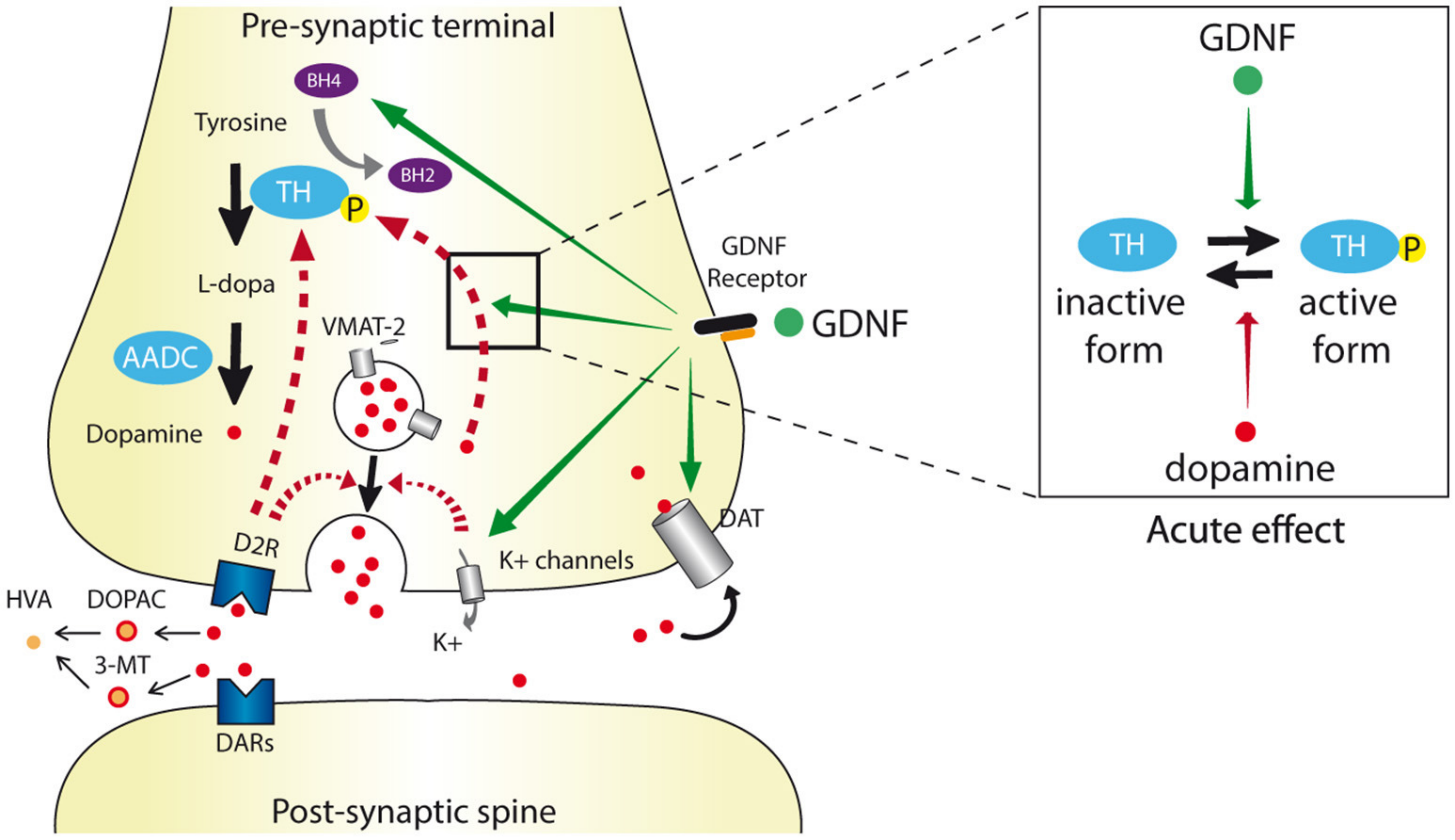
**Schematic dopaminergic synapse depicting the different levels of GDNF regulation**. The dopamine synthesis, release, re-uptake, and degradation pathways are indicated by black arrows. Dopamine is synthesized by conversion of tyrosine to L-DOPA by tyrosine hydroxylase (TH) that uses tetrahydrobiopterin (BH4) as a cofactor. L-DOPA is converted to dopamine by aromatic acid decarboxylase (AADC) and then integrated into pre-synaptic vesicles via the vesicular monoamine transporter 2 (VMAT2). After release into the synaptic cleft via exocytosis, dopamine acts on its receptors (DAR), is uptaken by the dopamine transporter (DAT) and degraded into 3-methoxytyramine (3-MT) and 3,4 dihydroxyphenyl acetic acid (DOPAC) leading to the final homovanillic acid (HVA) metabolite. Physiological negative feedback on dopamine release is indicated in hatched red arrows. GDNF alters dopaminergic transmission (orange arrows) by (i) increasing the BH4 levels, (ii) increasing Ca^2+^-evoked-dopamine release via inhibition of K+ channels and subsequent membrane depolarization, and (iii) reducing DAT activity. Depending of the GDNF dose and administration period, the ratio of TH and phosphorylated (active) TH levels can be either increased or decreased. Except for DAT regulation, the direct or indirect impact of the GDNF receptor complex (RET/GFRα1) on these herein described levels of regulation still need to be clarified.

DA is submitted to an intricate network of regulations controlling its homeostasis (see Figure 1). Firstly, DA regulates its own synthesis by binding to TH and retro-inhibiting its activity (Gordon et al., 2008). After release in the synaptic cleft, DA is rapidly re-uptaken by the dopamine transporter (DAT; Nirenberg et al., 1996; Uhl, 2003). Finally, binding to the pre-synaptic D_2_R autoreceptor results in a negative feed-back on DA synthesis and release (Westerink et al., 1994) whereas binding to D_1_R-type receptor on striatal medium spiny projection neurons provides a long-loop retro-control (Saklayen et al., 2004).

GDNF interferes with DA homeostasis at different levels (see Figure 1 and Table 1). It increases DA available in the synaptic cleft via different mechanisms such as: (i) stimulation of TH phosphorylation, which blocks the DA binding site and thus reduces the retro-inhibition of TH activity (Ramsey and Fitzpatrick, 1998; Gordon et al., 2008); (ii) GDNF enhancement of DA release via inhibition of a A type K^+^ channel thus provoking depolarization and Ca^2+^ entry (Hebert et al., 1996; Bourque and Trudeau, 2000; Yang et al., 2001); (iii) reduction of DAT activity via Ret/DAT interaction (Airavaara et al., 2004; Boger et al., 2007; Littrell et al., 2012; Barroso-Chinea et al., 2016). These effects can ultimately lead to compensatory mechanisms such as downregulation of TH transcription (Georgievska et al., 2002a).

**Table 1:** **GDNF-induced neurochemical changes in non-lesioned dopaminergic system**.

**Effects**	**Model**	**GDNF administration**			**References**
		**Time post-infusion**	**Infusion mode, doses**	**Injection site**	
**EFFECTS OF GDNF TREATMENT**					
Dose-dependent increase of DA uptake.	Rat midbrain culture	12 days	GDNF protein, 0.001–100 ng/mL	n.a.	Lin et al., 1993
Inhibition of transient A-type K+ channels leading to increased dopaminergic neurons excitability mediated by MAPK activation.	Rat midbrain slices	Acute	GDNF protein, 50 ng/mL	n.a.	Yang et al., 2001
Internalization of DAT leading to decreased DAT activity.	N2A cells overexpressing DAT and GFRα1	30 min	GDNF protein, 10 and 100 ng/mL	n.a.	Zhu et al., 2015
Direct interaction between RET and DAT.					
Basal extracellular and whole-tissue DA levels unchanged in the striatum. Evoked DA release increased after 3 but not 1 week. Increased extracellular DOPAC and HVA but unchanged whole-tissue levels at 3 weeks.	Healthy rat	1 and 3 Weeks	GDNF protein, 10 μg	SN	Hebert et al., 1996
Increased TH immunoreactivity in the striatum at 1 week.	Healthy rat	1 and 3 weeks	GDNF protein, 10 μg	SN	Hudson et al., 1995
Increased whole-tissue DA level in striatum and SN at 1 and 3 weeks. Increased HVA/DA whole-tissue levels in the striatum at 1 and 3 weeks (increased in SN at 1 week only).					
Increased TH phosphorylation on Ser31 and ERK2 phosphorylation in the striatum. Increased evoked-DA release.	Healthy rat	30 days	GDNF protein, 100 μg	Striatum	Salvatore et al., 2004
Decreased TH mRNA level in VTA / SN.	Healthy rat	13 months	Constitutive rLV-CMV-hGDNF 4.6 ng/mg tissue	Striatum	Rosenblad et al., 2003
Decreased TH immunoreactivity in striatum and SN. Striatal and nigral VMAT2 levels unchanged. Striatal DAT, D_1_R and D_2_R binding unchanged.					
Striatal TH immunoreactivity and protein levels unchanged at 3 weeks but decreased at 6, 12, and 24 weeks. Decreased TH activity: at 3, 6, 12, and 24 weeks. Unchanged DA tissue level at 1, 3, 6, 12 weeks and DOPAC level at 1, 3, 6 weeks. Increased HVA/DA tissue levels at 1, 3, 6 weeks. Unchanged VMAT2 immunoreactivity.	Healthy rat	3, 6, 12, and 24 weeks	Constitutive rLV-CMV-hGDNF 1.6–4.2 ng/mg tissue	Striatum^*^	Georgievska et al., 2004
Increased GTP cyclohydrolase (GTPCH I) activity and tetrahydrobiopterin (BH4) level in the striatum.	Healthy rat	3 months	Constitutive rLV-PGK-hGDNF not quantified.	Striatum	Sajadi et al., 2005
Decreased TH immunoreactivity and activity. Unchanged VMAT2.					
Decreased whole-tissue DA levels.					
Decreased TH immunoreactivity, significant at high GDNF doses only (219 and 338 pg/mg tissue).	Healthy rat	9 weeks	Inducible rAAV-V16-hGDNF, dose escalation 25 to 338 ng/mg tissue	Striatum	Chtarto et al., 2016
Decreased TH and phospho-TH at the highest GDNF dose only (253 pg/mg prot).	Healthy rat	5 weeks	Inducible rAAV-V16-70 and 253 pg/mg protein	Striatum	Barroso-Chinea et al., 2016
Decreased DA uptake, increased DAT dimerization and DAT/α-syn interactions at 70 and 253 pg/mg protein.					
DAT protein level unchanged at any dose.					
No DAT/D_2_R interaction.					
**EFFECT OF RET OR GDNF DEFICITS**					
Increased extra-cellular DA in Acc N. and striatum.	GDNF ± heterozygous mice	n.a	n.a	n.a	Airavaara et al., 2004
Increased DAT activity but striatal DAT protein level unchanged.	GDNF ± heterozygous mice	3 and 12 months	n.a	n.a	Boger et al., 2007
Increased DAT activity and intracellular DA in Acc N. but not in striatum.	Ret ± heterozygous mice	n.a.	n.a.	n.a.	Zhu et al., 2015

*SN, Substantia Nigra; DA, Dopamine; Acc N., Accumbens Nucleus; VTA, Ventral Tegmental Area; DOPAC, 3,4 dihydroxyphenyl acetic acid (DA metabolite); HVA, Homovanillic acid (DA metabolite); DAT, DA transporter; VMAT2, Vesicular monoamine transporter; TH, Tyrosine hydroxylase; D_1_R and D_2_R, D1 and D2 Dopaminergic Receptors; α-syn, α-synuclein; rLV, Recombinant Lentiviral Vector; rAAV, recombinant Adeno-Associated Viral Vector; hGDNF, human Glial cell-line–Derived Neurotrophic Factor; CMV, Cytomegalovirus promoter; n.a., non-applicable*.

* *In this study, a rLV-GDNF has also been injected into the SN, but it is not detailed in this table*.

Consequently, important variations of GDNF level are likely to profoundly perturb DA homeostasis. Whether a “normal” dopaminergic neurotransmission can be restored in the presence of supraphysiological GDNF concentrations cannot be predicted. Not surprisingly, long-term uncontrolled and sustained GDNF overexpression has led to compensatory changes (Georgievska et al., 2002b; Barroso-Chinea et al., 2016) that could have outweighed trophic effects. In contrast, a moderate GDNF overexpression is likely to compensate a diminished neurotrophic environment (Chauhan et al., 2001) and provide the possibility to re-establish a physiological DA homeostasis (Kumar et al., 2015). Indeed, a 60% increased GDNF level was sufficient to observe a protective effect toward a 6-OHDA lesion. Similarly, in other studies, neuroprotective effects in the absence of TH downregulation have been obtained by applying low GDNF doses either by injecting a low amount of viral vector (Eslamboli et al., 2005) or by controlling the level of transgene expression (Barroso-Chinea et al., 2016; Chtarto et al., 2016). Interestingly, using a discontinuous GDNF delivery paradigm in the partial rat 6-OHDA model, also allowed to reduce the behavioral symptoms as well as to maintain VMAT2-positive cells and innervation in the absence of TH downregulation (Tereshchenko et al., 2014).

### Pro-survival vs. neurochemical effects

Given the above-described GDNF-mediated multiple effects on dopaminergic neurotransmission, the mechanism of the neuroprotective effects observed in the numerous gene delivery studies (Bilang-Bleuel et al., 1997; Choi-Lundberg et al., 1997; Mandel et al., 1999; Kirik et al., 2000; Kordower et al., 2000) can be questioned. Whether neurons were protected against pro-apoptotic pathways or whether surviving but dysfunctional neurons were boosted to re-express lost markers is still an open question.

In addition to “waking-up” dysfunctional dopaminergic neurons, stimulation of striatal reinnervation in remaining healthy neurons (without increase of the number of cell bodies) is also a potential mechanism (Brizard et al., 2006). In particular, increased TH activity, through phosphorylation (Salvatore et al., 2004) and reduction of DAT activity (Barroso-Chinea et al., 2016) both leading to increased extracellular DA levels probably contribute, in addition to neuroprotection, to the observed behavioral benefits. In conclusion, several GDNF-mediated effects not related to its anti-apoptotic mechanism, could have biased the interpretation of behavioral and histological data (especially when TH alone was taken as a criteria; Sajadi et al., 2005; Yang et al., 2009; Barroso-Chinea et al., 2016).

### Cell type secreting GDNF

In the striatum, endogenous GDNF is expressed by parvalbumin-positive interneurons (Hidalgo-Figueroa et al., 2012). The different classes of viral vectors used to deliver GDNF [adenoviral vector (Choi-Lundberg et al., 1997; Connor et al., 1999; Kozlowski et al., 2000); lentiviral vectors (Bensadoun et al., 2000); and recombinant adeno-associated virus (rAAV)-based vectors (Mandel et al., 1999; Kirik et al., 2000)] transduce different cell types with varying efficiencies. The promoter used for transgene expression further influences the cell type specificity (Bockstael et al., 2008). In most studies, this issue has not been evaluated. Some vectors transduce the more abundant medium spiny neurons which project to the globus pallidus and SNr, thus resulting in an anterograde transport of the transgene product in these structures (Bockstael et al., 2008). In order to avoid this dissemination of GDNF in non-targeted structures which can provoke undesired effects (Manfredsson et al., 2009), some groups have directed GDNF expression into astrocytes in order to restrict transgene expression to the delivery site in the striatum (Do Thi et al., 2004; Pertusa et al., 2008; Drinkut et al., 2012). Considering the different cell type specificities of transgene expression mediated by the different vectors and the variable amounts of GDNF produced (see Table 1), it is very difficult to compare studies performed using different viral vectors.

Interestingly, mice which were manipulated to overexpress GDNF from the native locus (by deletion of miR-binding sites in the 3′ untranslated region) had an increased number of dopaminergic cells in the substantia nigra pars compacta (SNpc) as well as increased dopaminergic innervation and dopamine (DA) release in the striatum (Kumar et al., 2015). However, when these mice were treated with 6-OHDA, an aggravation of the DA level decrease in the striatum and dopaminergic neurons loss was observed. This surprising result was attributed to GDNF-mediated stimulation of DAT activity which increases 6-OHDA uptake. In order to evaluate GDNF neuroprotective effect using a toxin which does not depend on DAT, the authors injected lactacystin, a proteasome inhibitor which induces alpha-synuclein accumulation in nigral neurons. Although, motor symptoms were improved and dopaminergic levels were higher in GDNF hypermorphic mice the mutant mice, the number of dopaminergic neurons was not increased. As already outlined in Section Pro-Survival vs. Neurochemical Effects, this recent study further suggests that, due to the pleiotropy of GDNF functions, confounding factors might have biased the interpretation of putative pro-survival effects in previous studies.

## Neurotrophic factors gene delivery: still a promising clinical paradigm for parkinson's disease?

Clinical trials were conducted using catheters releasing recombinant GDNF protein (Nutt et al., 2003; Lang et al., 2006) as well as rAAV serotype 2 (rAAV2)-mediated delivery of the NRTN (Marks et al., 2010; Warren Olanow et al., 2015) or GDNF cDNA (https://clinicaltrials.gov/ct2/show/NCT01621581?term=AAV2-GDNF&rank=1). In the Phase I trials, AAV vectors were safe. However, the Phase II results were disappointing, although beneficial effects have been described for patients which have been followed for longer periods (Marks et al., 2010). Several factors could have reduced clinical benefits.

### Impact of axonopathy on NRTN signaling

The patients were enrolled in the AAV2-NRTN clinical trial at very late stages of the disease, usually more than 5 years post-diagnosis (Marks et al., 2010). Analysis of brains from untreated PD patients at different stages, from 1 to 37 years post-diagnosis, showed that the putaminal innervation, as measured by immunostaining against TH, had almost totally disappeared from 4 years post-diagnosis (Kordower et al., 2013). In contrast, numerous TH-positive dopaminergic neurons were still present in the SNpc. These data are in accordance with other studies pointing to a dying-back degeneration of dopaminergic neurons in PD. The current view that more than 80% dopaminergic neurons have died at the time of diagnosis has been revisited using new techniques to evaluate the number of surviving neurons and the putaminal dopaminergic innervation (using radioactive ligand binding to DAT; Burke and O'Malley, 2013; Kurowska et al., 2016). These data demonstrate that the extent of neuronal death at the time of symptoms onset is only 30% whereas putaminal DA levels were 50–70% reduced, suggesting that axon terminals become dysfunctional prior to cell death.

Post-mortem analysis of four patients enrolled in the AAV2-NRTN trial, showed that although surviving melanin-positive dopaminergic neurons were still present in the SNpc, TH-positive dopaminergic terminals in the putamen were very sparse and NRTN transport from the putamen to the SNpc was very slow and inefficient (Bartus et al., 2011, 2015). It should be noted that this analysis was based uniquely on TH immunohistochemistry. Thus, it cannot be excluded that dopaminergic projections were still present but did not express TH. Indeed, as already discussed above, TH expression does not always correlate with other dopaminergic markers, such as the vesicular monoamine transporter VMAT2 or aromatic acid decarboxylase (AADC; Bjorklund and Dunnett, 2007) and in some cases, fibers have lost TH expression but kept their ability for retrograde transport (Sauer and Oertel, 1994). Therefore, it cannot be excluded that the loss of TH expression observed in these patients could reflect a diseased state rather than the absence of putaminal innervation.

Why was NRTN not transported? A plausible hypothesis is that alpha-synuclein oligomers characteristic of early PD neuropathology (Schulz-Schaeffer, 2015), accumulating in the axons empede trafficking of the signaling endosomes. Indeed, in dementia with Lewy bodies, the greatest abundance of alpha-synuclein aggregates is found in the axons, more particularly in the pre-synaptic terminals (Kramer and Schulz-Schaeffer, 2007) causing synaptic pathology and loss of dendritic spines in the postsynaptic area.

Interestingly, alpha-synuclein fibrils have been shown to interfere with the trafficking of the BDNF/Trk-B signaling endosome (Watson et al., 1999; Volpicelli-Daley et al., 2014). Whether, like these neurotrophins, GFR–α ligands such as GDNF and NRTN exert their survival effect on dopaminergic neurons by a terminally-initiated signaling cascade is still unclear. Indeed, data obtained in compartmentalized cultures of sympathetic and dorsal root ganglia sensory neurons, suggested that GDNF pro-survival effect was predominantly related to a direct cell soma and a terminally-induced Ret-signaling, respectively, thus pointing to a cell type-specific GDNF protective mechanism (Tsui and Pierchala, 2010).

On the other hand, it has been suggested that in the absence of functional nigro-striatal fibers, GDNF expressed in striatal medium spiny neurons can be anterogradely transported to the SNpr and bind to its receptor in the neighboring SNpc, thus providing a trophic effect (Kells et al., 2010). However, other pre-clinical data have suggested that applying GDNF at the level of the SN could be deleterious either reducing the benefit of intrastriatal delivery (Kirik et al., 2000) or provoking local aberrant sprouting (Georgievska et al., 2002a). In accordance with these studies, in a subsequent clinical trial, two-sites rAAV2-NRTN delivery into both putamen and SNpc provided no clinical benefit (Warren Olanow et al., 2015).

Since the rAAV2 vectors used in the clinical trials transduce both interneurons and projection neurons, discriminating between a retrograde signaling mechanism and anterograde transport followed by a local signaling at the soma level has not been feasible. Interestingly, in the adult brain, GDNF is expressed by parvalbumin -positive interneurons (Hidalgo-Figueroa et al., 2012) which are distributed throughout the striatum in a topology which coincides with the distribution of dopaminergic neurons terminals. In this respect, GDNF delivery via AAV2-mediated gene transfer does not recapitulate the physiological GDNF secretion.

Strategies targeting separately either interneurons or striato-nigral projection neurons could help to unravel the mechanism of the neurotrophic effect and design more promising therapeutical approaches.

Finally, if the nigrostriatal projections are not simply dysfunctional but degenerated and thus absent from the putamen, it is likely that terminally-administered neurotrophic factors will fail to rescue a functional nigro-striatal pathway.

### Lack of predictability of pre-clinical animal models?

The clinical data were not predicted by the pre-clinical animal models generated by the Ceregene group. Indeed, in the acute MPTP-induced macaque model described by Bartus and collaborators (Bartus et al., 2011), the surviving nigro-striatal dopaminergic neurons still had functional projections, proficient for retrograde transport. In contrast, in the patients samples, <1% of surviving melanin-positive neurons in the SNpc were co-staining with NRTN at 1.5 and 3 months post-surgery and about 5% at 4 years post-surgery.

These data emphasize the need to perform pre-clinical studies in animal models which recapitulate the progression of the pathology taking into account recent basic research. Indeed, accumulating evidence suggest that PD neurodegeneration is initiated in axon terminals (Schulz-Schaeffer, 2015). Several authors reported neuropathological hallmarks matching with this mechanism in animal models. Indeed, in a chronic and progressive MPTP-induced macaque model described by the Bezard's group, at the mean onset of Parkinsonian symptoms, striatal DAT binding and DA content decreased to, respectively, 20 and 18% of untreated monkeys while 57% of nigral TH-positive neurons were spared (Meissner et al., 2003).

Interestingly, the Krystof Bankiewicz group has compared the effects of intraputaminal AAV2-GDNF injection in a mild and an almost complete MPTP lesion in the macaque (Kells et al., 2010). They have evidenced functional improvements (as evidenced by positron emission tomography with 6-[18F]fluoro-l-m-tyrosine scans) and increased TH putaminal innervation and SNpc labellings with both types of lesions. The dopaminergic activities and TH-positive fibers increase in the nearly complete lesion paradigm, were significantly increased but nevertheless only localized in a restricted region of the putamen. These data are in accordance with the post-mortem analysis of patients at 4 years post-injection of rAAV2-NRTN, in which, despite the nearly total absence of TH staining in the putamen, a TH-positive innervation appeared in a limited area in the vicinity of the vector injection (Bartus et al., 2015).

In addition to phenotypic toxin-induced models, genetic models based on the transgenic expression of mutants isolated in familial PD cases provide valuable tools to evaluate gene therapy approaches. Notably, in a virally-mediated local alpha-synuclein transgenes is, as observed in patients populations (Kordower et al., 2013), axonopathy preceeded neuronal cell death (Garcia-Reitbock et al., 2010; Van der Perren et al., 2015). Intriguingly, GDNF had no effect on dopaminergic neuron survival and motor symptoms in two different local, intranigral alpha-synuclein transgenic models: a lentiviral vector mediated expression of the human A30P mutant in mice (Lo Bianco et al., 2004) and a rAAV-mediated overexpression of human wild-type alpha-synuclein in rats (Decressac et al., 2011). This failure has been attributed to Ret downregulation and disruption of GDNF signaling due to Nurr-1 downregulation induced by alpha-synuclein overexpression (Decressac et al., 2012). However, it should be noted that, in contrast to this report, two other studies showed that neither RET protein, nor Ret mRNA are downregulated in patients with Lewy bodies (Walker et al., 1998; Backman et al., 2006). It might be that the concentration of intranigral alpha-synuclein protein expressed by the viral vectors was higher than in PD patients and induced collateral effects (Hoffer and Harvey, 2011). The quantitative evaluation of alpha-synuclein content in Parkinson's disease patients post-mortem tissue has proven difficult due to the existence of multiple alpha-synuclein forms and difficulties in solubilizing these proteins. In one study, membrane-associated sodium dodecyl sulfate soluble full-length 17 kDa and high molecular weight alpha-synuclein species were only slightly increased in PD patients as compared to healthy subjects (Tong et al., 2010) whereas in another study total alpha-synuclein was suggested to be 11-fold increased in fresh frozen protein extracts (Shehadeh et al., 2009). Since the proportion of monomeric vs. multimeric alpha-synuclein species as well as their conformation, which play an important role in toxic effects (Peelaerts et al., 2015), were not quantified separately, it is difficult to evaluate the predictive value of the animal models.

In the study by Decressac et al. (2011, 2012) the alpha-synuclein concentration in the substantia nigra of the rAAV-injected rats has not been reported. In other studies using rAAV vectors, the alpha-synuclein levels were found to be three- to four-fold increased (Gorbatyuk et al., 2010; Landeck et al., 2017). However, since the AAV vector serotype, the viral preparation titer and the promoter used for transgene expression differed from those used in the study by Decressac et al. (2011, 2012) these data cannot possibly be compared to the patients data. In addition, it has recently been shown that in contrast to virally-delivered human alpha-synuclein, rat alpha-synuclein induced no detectable neurodegeneration at similar vector doses. These data are questioning the conclusions of studies using human alpha-synuclein in rodent models.

Physiologically, alpha-synuclein-mediated SNARE-complex assembly is necessary for synaptic function but at high doses, alpha-synuclein pathologically misfolds into neurotoxic forms. Accordingly, alpha-synuclein has been shown to inhibit synaptic vesicle exocytosis in transfected midbrain dopaminergic neurons cultures in a dose-response manner (Burre et al., 2010; Lundblad et al., 2012). Toxic alpha-synuclein fibrils was also shown to impair the retrograde transport of BDNF signaling endosome (Volpicelli-Daley et al., 2014). Whether, in the study by Decressac et al. (2011), alpha-synuclein overexpression (Nemani et al., 2010), in addition to reducing the Ret signaling cascade (Decressac et al., 2012) also resulted in (i) a reduction of DA release and re-uptake and/or (ii) blocked GDNF retrograde transport has not been investigated (Lundblad et al., 2012; See also reference (Hoffer and Harvey, 2011) for an extensive discussion about the potential pitfalls of the rAAV-mediated alpha-synuclein model for the evaluation of the therapeutical effect of GDNF gene delivery). Interestingly, injection of pre-formed α-synuclein fibrils seemed to faithfully recapitulate the hallmarks of the pathology (Volpicelli-Daley et al., 2014, 2016).

Finally, transgenic mice harboring a mutant LRRK2 gene (the most frequent mutation in PD) also provides an interesting, potentially clinically-relevant, phenotype. Indeed, in this model no loss of dopaminergic neuron cell bodies was observed, whereas axons harbored dystrophic neuritis (Li et al., 2009).

### Neurturin vs. GDNF

Pre-clinical studies established the neuroprotective potential of NRTN gene delivery (Fjord-Larsen et al., 2005; Kordower et al., 2006; Gasmi et al., 2007a,b; Herzog et al., 2009). However, the amount of basic knowledge about NRTN is far less abundant than for GDNF and outlines important differences between the two neurotrophic factors.

First, the induction of Ret-mediated signaling is expected to be far less efficient after NRTN as compared to GDNF gene delivery. Indeed, dopaminergic neurons express GFR-α1 but not GFR–α2, the preferred NRTN primary receptor. NRTN can bind to GFR-α1 but with a much lower affinity than GDNF (Kramer and Liss, 2015). In addition, NRTN diffuses less efficiently than GDNF in the parenchyma (Runeberg-Roos et al., 2016). Finally, contrarily to GDNF, NRTN endogenous secretion signal is weak and in order to reach efficiency, in the genetic construct developed for gene therapy, it has been replaced by a mice immunoglobulin signal peptide (Fjord-Larsen et al., 2005).

### Delivery issues: poor coverage of the target structure

The rAAV2 viral particles (Nguyen et al., 2001; Burger et al., 2004; Hadaczek et al., 2006; Lubansu et al., 2008) as well as NRTN itself (Bespalov et al., 2011) poorly diffuse in the brain parenchyma. Post-mortem analyses showed that only ~15% of the putamen was covered with NRTN (Bartus et al., 2011), which could be suboptimal to observe a significant therapeutical effect. Therefore, interpretation of clinical and histopathological data from the rAAV2-NRTN clinical trials should be taken with caution.

NRTN binds to heparan sulfate, which severely reduces its diffusion (Bespalov et al., 2011). Recently, NRTN mutants (mutated in heparan sulfate binding sites) were shown to diffuse further away from the delivery site and to be more neuroprotective in the 6-OHDA model (Runeberg-Roos et al., 2016). The use of optimized neurosurgical techniques could further help overcoming this limitation (Johnston et al., 2009).

### Delivery issues: long-term uninterrupted GDNF treatment induces compensatory effects

In preclinical models, compensatory effects reducing the expression of enzymes of the DA biosynthesis pathway (Georgievska et al., 2002a; Chtarto et al., 2007) as well as of DAT activity (Barroso-Chinea et al., 2016) appeared after long-term continuous treatment with GDNF. These neurochemical effects are likely to interfere with neurotrophic effects and possibly reduce clinical benefits. Interestingly, pulses of GDNF delivery provided similar neuroprotection as a continuous treatment while avoiding TH downregulation (Tereshchenko et al., 2014). Therefore, repeated short-term expression rather that continuous GDNF administration might constitute the treatment of choice for PD.

## Time to revisit GDNF gene therapy paradigm?

### Taking into account early axonopathy

Novel knowledge indicates that PD neurodegeneration is initiated at the level of the terminals and that neuron cell death is a remote consequence of synaptic dysfunction rather than a primary event. Therefore, whether GFR-α ligands in complex with Ret, activating anti-apoptotic and neurotrophic signaling cascades, constitute a relevant disease-modifying tool could be questioned.

In advanced patients (from 5 years post-diagnosis), TH-positive putaminal innervation has almost completely disappeared (Kordower et al., 2013). rAAV2-NRTN administered at least 5-years post-diagnosis (Marks et al., 2008) failed to provide a clear significant clinical benefit (Marks et al., 2010). In a subsequent clinical trial combining intraputaminal and intranigral vector administration, less advanced patients were included (Warren Olanow et al., 2015). A *post-hoc* analysis including the patients into subgroups according to the advancement of the disease at the time of surgery, seemed to indicate that the therapy could have benefited to less advanced patients (Bartus and Johnson, 2017a,b).

### Effects of GDNF on dopaminergic neurotransmission

As outlined above, GDNF mediates neurochemical effects either augmenting the dopaminergic function or inducing compensatory mechanisms reducing the dopaminergic function. These effects may play an important role in the observed beneficial effects on motor symptoms in pre-clinical studies and possibly in clinical studies (Marks et al., 2010; Bartus et al., 2014). Further studies dissecting the mechanism of specific GDNF functional effects are required in order to interprete the clinical data and possibly design new viral vectors and clinical protocols fully exploiting the neuroprotective effects of GDNF while avoiding confounding effects.

### Avoid undesirable compensatory effects of sustained GDNF administration at supraphysiological doses

Except in one study (Eslamboli et al., 2005), in which GDNF striatal concentration was only three-fold higher than the endogenous level, in most preclinical studies it was increased at least 10-fold (Kirik et al., 2000; Georgievska et al., 2002a; Yang et al., 2009). In such conditions of excessive GDNF overexpression, time-dependent compensatory mechanisms affecting both the motor behavior and DA biosynthesis and turn-over were observed (Kirik et al., 2000; Georgievska et al., 2002a; Yang et al., 2009). Therefore, GDNF administration should be adjusted to a concentration which does not perturb DA homeostasis and the treatment should be interrupted before the appearance of compensatory effects.

### Physiological GDNF secretory pathway

GDNF secretion can either be constitutive or regulated by depolarization and Ca^2+^ entry (Lonka-Nevalaita et al., 2010). In the rodent striatum, GDNF is natively expressed by parvalbumin interneurons (Hidalgo-Figueroa et al., 2012; d'Anglemont de Tassigny et al., 2015) which are fast spiking neurons, controlling the activity of medium spiny neurons of the direct and indirect pathways and closely associated with dopaminergic neurons terminals (d'Anglemont de Tassigny et al., 2015). None of the viral vectors used so far provides a targeted transgene expression into parvalbumin interneurons.

### Future directions

Although, the results of clinical trials using GDNF recombinant protein or NRTN gene delivery for PD patients have so far been disappointing, evolving basic knowledge on PD physiopathology and GDNF biology as well as improvements of viral vectors technology justify pursuing neuroprotective approaches. However, understanding the respective dose range, kinetics and cellular specificity of neurotrophic and neurochemical effects and adapting the amounts of neurotrophic factor administered and the periods of treatment will be of utmost importance for the success of this emerging disease-modifying treatment.

Regardless, the recent data showing that TH-positive putaminal innervation has almost completely disappeared at 5 years post-diagnosis, questions the feasibility of treating advanced patients with neurotrophic factors. Hopefully, the demonstration of the safety of rAAV administration into the human brain (Kaplitt et al., 2007; Marks et al., 2008; LeWitt et al., 2011; Leone et al., 2012; Bartus et al., 2013; Tardieu et al., 2014; Warren Olanow et al., 2015) will pave the way for new trials enrolling less advanced patients.

## Author contributions

LT wrote the review and critically read the table and figure. MH made the table and the figure and critically read the manuscript.

### Conflict of interest statement

The authors declare that the research was conducted in the absence of any commercial or financial relationships that could be construed as a potential conflict of interest.
